# Considerations on the Assessment and Use of Cycling Performance Metrics and their Integration in the Athlete's Biological Passport

**DOI:** 10.3389/fphys.2017.00912

**Published:** 2017-11-09

**Authors:** Paolo Menaspà, Chris R. Abbiss

**Affiliations:** Centre for Exercise and Sports Science Research, School of Medical and Health Sciences, Edith Cowan University, Joondalup, WA, Australia

**Keywords:** antidoping, performance monitoring, biometrics, cycling performance, accuracy

## Abstract

Over the past few decades the possibility to capture real-time data from road cyclists has drastically improved. Given the increasing pressure for improved transparency and openness, there has been an increase in publication of cyclists' physiological and performance data. Recently, it has been suggested that the use of such performance biometrics may be used to strengthen the sensitivity and applicability of the Athlete Biological Passport (ABP) and aid in the fight against doping. This is an interesting concept which has merit, although there are several important factors that need to be considered. These factors include accuracy of the data collected and validity (and reliability) of the subsequent performance modeling. In order to guarantee high quality standards, the implementation of well-structured Quality-Systems within sporting organizations should be considered, and external certifications may be required. Various modeling techniques have been developed, many of which are based on fundamental intensity/time relationships. These models have increased our understanding of performance but are currently limited in their application, for example due to the largely unaccounted effects of environmental factors such as, heat and altitude. In conclusion, in order to use power data as a performance biometric to be integrated in the biological passport, a number of actions must be taken to ensure accuracy of the data and better understand road cycling performance in the field. This article aims to outline considerations in the quantification of cycling performance, also presenting an alternative method (i.e., monitoring race results) to allow for determination of unusual performance improvements.

## Introduction

Live broadcasting of performance metrics (e.g., power output, speed, and cadence) during road cycling competitions has become a reality in modern times (i.e., starting in 2016). Sharing performance data can be a great way to increase fan engagement and facilitate promotion and development of the sport. Recently, as a result of increasing public and media pressure for transparency and openness (Tucker and Dugas, 2015), physiological data of several professional cyclists has been published, including data from a multiple Tour de France winner (Bell et al., 2017). Together with the increase in transparency and sharing of performance metrics, it has been suggested that performance and physiological biometrics may strengthen the sensitivity and applicability of the Athlete Biological Passport (ABP) in the fight against doping (Tucker and Dugas, 2015).

It should be noted that attempts of using physiological data to legitimate extraordinary cycling performances is not new in cycling. In fact, laboratory data of multiple Tour de France winner Lance Armstrong were published in a high impact factor peer-reviewed journal in order to characterize his extraordinary physiology (Coyle, 2005). The publication of this manuscript is important within this context given that, as per late admission by the author, the outcomes of the study may have been influenced by the cyclist's drug use (Coyle, 2013). This admission highlights that it is almost impossible to be absolutely certain about the use, or not, of doping from indirect evidence like the observation of physiological data. Additionally, it is also important to note that the methods and conclusions within this study have been heavily critiqued (Martin et al., 2005; Gore et al., 2008), which outlines the caution that needs to be taken with the use of such data. Indeed, the idea of using performance biometrics in association with the ABP has been primarily criticized due to the fact that, given a degree of imprecision and associated doubt (Tucker and Dugas, 2015; Gleaves, 2017), it would not be fair and ethical to use these data to “raise questions” (Gleaves, 2017). This manuscript outlines several considerations in the assessment of competitive cycling performance metrics with consideration to the ABP.

## Accuracy of power meters

With the rapid expansion of various cycling power meters on the market, it has become increasingly challenging for timely independent assessment of the accuracy of such devices. At present, there are several different brands and models of power meter available, which may be placed on different locations of the bike, including the rear wheel, crank arms (or single arm), pedals/cleats, or bottom bracket/axle. Of note, the power meter placement in itself influences the power output recorded, for example power measured at the cranks is higher than power measured at the rear hub due to frictional losses in the drivetrain (Passfield et al., 2017).

To date, several studies have investigated the accuracy of the various existing cycling power meters. Depending on the specific study protocol, the range of power outputs investigated and the brand/model, results vary with errors up to 16.5% (Gardner et al., 2004; Bertucci et al., 2005; Hurst and Atkins, 2006; Duc et al., 2007; Maier et al., 2017). While some individual devices have been shown to be inaccurate shortly after manufacturing (Gardner et al., 2004), other factors difficult to control, such as, ambient temperature, vibrations, and impacts from gear shifts, but also bicycle and power meter maintenance have been suggested as variables influencing the accuracy of power assessment (Gardner et al., 2004; Maier et al., 2017; Passfield et al., 2017). For the above mentioned reasons, the consistent accuracy of commercial power meter devices cannot not be presumed. Complicating this issue is the fact that several different methods have been used to validate or calibrate cycling power meters, including static calibration, dynamic calibration, and comparison with gold-standard devices (Gardner et al., 2004; Bertucci et al., 2005). It is important to note that different calibration procedures may convey different results and as such the need for a standardization of calibration methodologies has been highlighted (Hawser, 2016). In this regard, the use of first principle-based calibration methods are recommended (Stein et al., 1967; Maier et al., 2017).

## Quality system and certification

A possible partial solution to the calibration issues mentioned in the previous paragraph could be the use of external quality control organizations (certification bodies) to either validate the equipment used or certify that a quality management system is in place (Tanner and Gore, 2013). If this were the case, professional cycling teams and National federations would be required to seek appropriate certification (such as, ISO 9001 or similar) (Guasch et al., 2007; Tanner and Gore, 2013). The certification process, requiring internal and external audits, would not be able to guarantee the absence of malicious data manipulation (e.g., change in calibration slope to artificially manipulate power reading), but nevertheless, it could be a required step to guarantee a minimum standard of quality. Importantly, however, such quality insurance would incur significant costs, and in addition to the costs of the power meter devices themselves, it would likely inhibit such monitoring in many cycling organizations, particularly at junior or developmental levels. As such, this performance passport would not be useful in identifying early cheating, limiting its effectiveness in the prevention of doping. A possible solution to this problem could be the use of “neutral” power meters (e.g., pedals provided by the Union Cycliste Internationale) that directly store and encrypt measured data. These power meters could be distributed to a certain number of riders at each race, some randomly selected athletes and some athletes chosen because of their recent performances (i.e., similarly to what happens in anti-doping testing).

## Field data and performance modeling

In the “Age of the Twittersphere” (Burke, 2017), it is becoming increasingly common to find online estimations of cycling power output based on available data such as, climbing time, elevation gain, and environmental conditions. Unfortunately, these estimations are likely to have an error of between 6 and 10% (Millet et al., 2014). Given some level of skepticism of the general public that the cycling peloton is entirely “clean,” online dissemination of inaccurate estimations are likely to be damaging to an athlete's career. Regardless, the detailed analysis with these estimations and the associated debates on social media highlight the public's interest in such data. As a result, the collection and dissemination of accurate performance data may not only be important in the context of anti-doping, but also in improving viewership and marketing of the sport. Regardless of the context in which such data is collected, there is need for an organized structure to monitor performance, which would contribute in improving the integrity of the sport.

In order to properly use power output data for performance modeling, or as a performance biometric to be integrated in the ABP, there are several important considerations in the analysis and interpretation of such data. Firstly, there is no single reference measurement of cycling performance (Jobson et al., 2009; Passfield et al., 2017), with several approaches used to monitor and evaluate competitive cycling performance data, including but not limited to the assessment of critical power (Karsten et al., 2014; Skiba et al., 2014), maximal mean power (Ebert et al., 2005; Quod et al., 2010), and record power profile (Pinot and Grappe, 2011, 2015). Additionally, it is extremely important to know if the performances were truly maximal. For this reason a challenge in developing a typical performance profile of any athlete's training and racing data may be the various tactics within road cycling. Indeed, some cyclists may rarely reach maximal short duration intensities during a race (i.e., *domestiques*), especially if their role is to “control” the first section of a race therefore producing very long efforts at submaximal workloads before aiming to minimize workload for the remainder of a race (Rodriguez-Marroyo et al., 2009). Furthermore, the historical focus of performance modeling within cycling has been on endurance based activities or tasks (i.e., uphill cycling and time trials). For this reason, particular attention needs to be given to other cycling tasks such as road sprinting. Given the specific characteristics of sprinting, the traditional “power-duration” methods used to assess endurance cycling performances may not be optimal for longitudinal monitoring of changes in anaerobic performance metrics of road sprinters. Furthermore, optimal quantification of cycling performance would benefit from accurate assessment of the cyclists' total body and equipment weight (Gleaves, 2017), particularly during uphill cycling (Millet et al., 2014). In theory such assessment could be possible prior to or following cycling competitions but it may be difficult to obtain during competitions and it could be source of additional measurement errors.

Additional to technical and tactical factors, there are several important biomechanical and physiological factors that influence one's ability to generate power. For instance, it has been shown that the position of the cyclist on the bike has a significant effect on power production, with lower torso angle impairing power production (Fintelman et al., 2014, 2015). As such, cycling data recorded on road bikes (relatively high torso angle) would need to be compared separately to time trial performance (relatively low torso angle). Similarly, the ability to produce power seems to be influenced by the road slope, with cyclists generating greater power output while riding uphill when compared to flat terrain (Sassi et al., 2005; Nimmerichter et al., 2012). These effects are observed on mountainous compared with flat stage races, despite the negative influence of altitude on cycling performance. Indeed, environmental conditions such as hypoxia and heat impair power production and their effects would need to be considered when comparing cycling performances (Nybo, 2010; Garvican-Lewis et al., 2015; Townsend et al., 2017). While research has attempted to predict the effects of acute hypoxia on exercise capacity (i.e., critical power), the effects of environmental heat on performance is not immediate and complicated by many factors including convective cooling, blood distribution, core, skin, and environmental temperature gradients. Indeed, fatigue development is incredibly complex and has obvious implications for the quantification of performance (Abbiss and Laursen, 2005; Van Cutsem et al., 2017). Consequently, careful consideration should be given when comparing performances in 1-day events (e.g., World Championships) or at the end of 3-week stage races (e.g., Tour de France; Rodriguez-Marroyo et al., 2017). As such, it is not currently possible to compare performance data recorded across the range of cycling events that athletes may compete in. It is also important to note that although professional cyclists compete regularly, many cyclists may aim to reach peak performance in few competition per year or each 4 years in the case of an Olympic cycle. Literature examining the longitudinal changes in cycling performance or load is currently scarce (Sassi et al., 2008; Metcalfe et al., 2017).

In order to use measurements of power output as a performance biometric to be integrated in the ABP, an in depth understanding of what could be considered normal would be necessary. A considerable degree of research would be required in order to build evidence of what are the normal seasonal (Sassi et al., 2008) and longitudinal changes (Pinot and Grappe, 2015), in men and women's cycling. Furthermore, research is needed in order to outline the typical rate of performance improvement in cyclists transitioning from development categories to the professional rankings (Menaspà et al., 2013; Pinot and Grappe, 2015), as well as, performance decline associated with aging (Capelli, 2017). Particular attention is needed examining the typical performance characteristics of exceptional professional cyclists (Mujika, 2012).

## A possible alternative

Cycling is an extremely complex sport and although it is well accepted that enhancement in an athlete's physiology increases exercise capacity, the effect of such changes on overall race performance has not been well-investigated (Plumb et al., 2016). Regardless, it seems reasonable to assume that the most direct goal of doping is to improve race results. For this reason, directly monitoring race results (e.g., UCI points, Filipas et al., 2016, 2017) may be an alternative or additional mechanism by which to better understand normal and abnormal performance trends. Of course, such analysis has limitations and would require considerable research to establish typical performance trends. An important disadvantage of this approach is that race results are only relative to the performances of all the other riders. Given the revelations about the possible high prevalence of doping in elite sport (Ulrich et al., 2017), race results of a high percentage of athletes could be heavily biased. Conversely, an advantage of this approach is that a database already exist and previous results could be examined. Furthermore, directly monitoring performance outcomes—rather than an athlete's capacity—has the additional benefit of recognizing unusual performance improvements otherwise undetectable using power output or the ABP, such as, mechanical doping.

## Conclusions

In Conclusion, there has been a rapid increase in the capacity to capture high resolution, real-time data from road cyclists. This data has been important in understanding the performance characteristics and demands of competitive road cycling and is not only utilized in professional settings but has also become available to the general cycling community. It has recently been suggested that the transparent dissemination of individual athletes' performance data may be used to better inform the fight against doping. It appears that a cyclist's power output may be the key performance metric to be monitored. However, while the assessment of power output may be common, there are several important aspects regarding the validity, reliability, and analysis of performance metrics that require careful consideration. The use of power data as a performance biometric within the ABP has merit but, based on the limitations discussed in this manuscript, it is currently not recommended. Regardless, such data is becoming increasingly influential within the development of cycling and as a result careful consideration is needed in order to ensure that the collection of such data improves rather than undermines the integrity of the sport.

## Author contributions

PM: Substantial contributions to the conception of the work, drafting the work, final approval of the version to be published, and agreement to be accountable for all aspects of the work. CA: Substantial contributions to the conception of the work, revising the work critically for important intellectual content, final approval of the version to be published, and agreement to be accountable for all aspects of the work.

### Conflict of interest statement

The authors declare that the research was conducted in the absence of any commercial or financial relationships that could be construed as a potential conflict of interest.
